# Evaluation of Perceptions of Tobacco Cessation among the Individuals Attending a Tertiary Care Dental Hospital – A Mixed Methods Design

**DOI:** 10.31557/APJCP.2021.22.9.2749

**Published:** 2021-09

**Authors:** Priyanka Ravi, Anupama Ivaturi, Diptajit Das, Upendra Singh Bhadauria, Charu Khurana, Monica Dev, Harsh Priya

**Affiliations:** 1 *Division of Public Health Dentistry, Centre of Dental Education and Research, All India Institute of Medical Sciences, New Delhi, India. *; 2 *National Oral Health Program, Centre for Dental Education and Research, All India Institute of Medical Sciences, New Delhi, India. *

**Keywords:** Tobacco, Cessation counseling, Tobacco quitting, Quitting perception- India

## Abstract

**Introduction:**

In India every year, an estimated 1 million people die from tobacco-related illnesses, which could have been avoided by stopping tobacco use. This study aimed to determine the tobacco dependence and perceptions about tobacco cessation among the patients attending a tertiary care dental hospital.

**Methods:**

A mixed-methods study was conducted among the individuals visiting the dental hospital. Self-administered questionnaires were used to record quantitative data on the current and past tobacco history, tobacco dependence, and quit attempts. Face-to-face interviews were conducted to collect qualitative data on the perceptions on tobacco quitting.

**Results:**

About 52% of participants had made an attempt to quit tobacco in the past. The majority of the smokers (82.7%), smokeless tobacco (SLT) users (71.3%), and dual users (83.1%) reported being in the contemplation phase of quitting tobacco. SLT users (12.3%) reported to have taken action in the past to quit tobacco compared to 9.6% smokers, and 1.4% of dual users. Doctors advise, motivation from family and friends motivated the participants to quit tobacco. The risk perception of tobacco use was reported as death, cancer, dental diseases, systemic diseases like chest congestion, cough, tuberculosis, lung cancer, and general body weakness. However, some of the participants were unaware of the methods to quit tobacco, some had misconceptions that shifting to other forms of tobacco and alcohol could help in quitting, and considered SLT to be less harmful than smoking forms.

**Conclusion:**

Majority of the participants were willing to quit tobacco and had taken measures to quit in the past. There is a need to increase the awareness among the tobacco users about the health risk of tobacco use, and motivate them to utilize the existing cessation services available.

## Introduction

Tobacco use is the leading single preventable cause of death worldwide and each year an estimated seven million deaths are attributed to the use of tobacco (WHO, 2020). According to the Global Burden of Disease (GBD) (2015), tobacco use contributed to 5.9% of total Disability-Adjusted Life Year (DALYs) in India. India is the third-largest tobacco producing nation and second-largest consumer of tobacco world-wide, and mortality due to tobacco is estimated at upwards of 1.3 million annually (Gupta et al., 1999; Sinha et al., 2014). 

According to the Indian Global Adult Tobacco Survey (GATS) 2, (2017) 28.6 % of adults use tobacco in some form, 24.9% are daily tobacco users and 3.71% are occasional users. Nearly two in five smokers attempted to quit smoking and one-third of smokeless tobacco (SLT) users attempted to quit in the past 12 months. From GATS 1 to GATS 2 the proportion of smokers and SLT users who were interested or planning to quit smoking had increased significantly from 46.6 to 55.4 and 45.2 to 49.7 respectively (GATS 2, 2017). The wave 1 of the International Tobacco Control Policy Evaluation (TCP) India Survey reported that one-fifth of tobacco users in India intended to quit tobacco use (Dhumal, 2014). 

The quitting perception among the tobacco users are influenced by various factors, Siddiqui et al., (2021) reported even though SLT users were highly motivated to quit yet were unsuccessful due to socio-environmental factors, persistent driving factors to continue consumption and lack of formal cessation resources and support. Pattanayak et al., (2012) studied the perceived health risk, and readiness to quit and reported inadequate knowledge, low-risk perception, casual attitude for tobacco-related health risks, and higher perceived ability to quit tobacco was seen among the bipolar disorder patients. Lack of available support for tobacco cessation, leading to failure in quitting, a lack of understanding about the ill effects of tobacco and an overall lack of belief in the existing tobacco control measures were some of the perceptions of tobacco use among the youth of India (Ghose et al., 2019). Delbarre and Aghi (2013) conducted a qualitative study among the vulnerable population of India and France and reported that the study participants anticipated quitting to be extremely challenging, and especially the Indian participants had negative perception towards quitting and believed smokeless tobacco actually improved their health. 

A Cochrane systematic review has reported that behavioral interventions for tobacco cessation conducted by oral health professionals in the dental office or community setting may increase tobacco abstinence rates among tobacco users (Carr and Ebbert, 2012). Ministry of Health and Family Welfare, Government of India (2018) had released the operational guidelines for establishing the tobacco cessation centers at the dental institutions. India dental graduates are aware of the of 5A’s and 5R’s protocol for tobacco cessation counseling (Kachwaha et al., 2019), and tobacco users are receptive towards tobacco counseling and services in the dental setting (Patil et al., 2015). Therefore, we should leverage the dental setting to understand patient perception of tobacco cessation and construct tailored cessation counseling for smoking, SLT, and dual (using both smoking and SLT) tobacco users.

The tobacco cessation centers have been functioning in the dental institutions of India, it is vital for the dental professionals to understand the various factors involved in tobacco quitting perception. Hence, this study was conceptualized to determine the tobacco dependence and perceptions of tobacco cessation among the individuals attending a tertiary care dental hospital. The objectives are to qualitatively determine their perception on tobacco use and cessation, and quantitatively determine the tobacco use, dependence, motivation and past quit attempts among the tobacco users. The mixed-methods study design was chosen because of its potential to generate more complete and higher quality data in an area that has not been sufficiently studied (Hadi et al. 3013).

## Materials and Methods

A mixed-methods study was conducted among the individuals visiting the dental hospital for duration of three months. 

The study was conducted in the following phases: phase I was qualitative and phase II was quantitative.

i) Phase I (Qualitative): The qualitative data aimed to capture the perception of tobacco use and cessation. A total of 56 in-depth interviews were conducted based on constructivist grounded theory methodology. Purposive sampling technique was used to recruit the participants. Interviews were conducted until data saturation was achieved and until patterns were captured related to study objectives. It was conducted by two qualified trained dentists. An interview guide was developed by all the investigators to determine the quitting perception. The responses were recorded in the form of interviewer notes by two trained investigators and were expanded later. Each interview lasted for around 20 to 30 minutes. No incentives were provided for the participants. These responses were subsequently coded and then eventually categorized.

ii) Phase II (Quantitative): The quantitative data was collected to determine the tobacco use, dependence and tobacco quitting practices among the study participants. A semi-structured questionnaire was administered to record the history of current and past tobacco use, tobacco quitting attempts in the past, tobacco dependence, and motivation to quit tobacco. The quit attempt was operationally defined as an individual making an attempt to quit tobacco and not using any form of tobacco for atleast 24 hours. The tobacco dependence was measured using The Fagerström Test for Nicotine Dependence (FTND) and The Fagerström Test for Nicotine Dependence-Smokeless Tobacco (FTND-ST) (Fagerstrom et al., 1992; Ebbert et al., 2006). The dual users filled both FTND and FTND-ST. The tobacco user’s motivation to quit tobacco was assessed using the Transtheoretical Model (Stages of Change) i.e., precontemplation, contemplation, preparation, action, maintenance and termination (Prochaska et al., 1992).

The inclusion criteria were the current tobacco users defined as those who were using any form of tobacco at present and had quit any form of tobacco in less than 3 months, individuals who were above 18 years of age and those willing to give written informed consent. Past tobacco users were those who had quit tobacco before 3 months and those who could not communicate verbally in English or Hindi languages were excluded. 

Informed consent was obtained from all the study participants. Ethical approval was obtained from the Institute Ethics Committee All India Institute of Medical Sciences, New Delhi. 

The validity and reliability of the self-administered questionnaire were tested by a pilot study on 10 respondents who were representative of the study subjects but not included in the study (k =0.85). 

*Statistical analysis*

*Qualitative data analysis*

All data from interviews were organized using ATLAS.ti software. A thematic analysis was used on the qualitative data gathered from the study participants as suggested by Braun and Clark (2006). The coding was done by reading all transcripts by two independent reviewers. Patterns from the data were generated and then organized as per similar attribute and used for creating themes. Similar themes were then compared and contrasted to create overarching themes. 

*Quantitative data analysis*

The data was entered in the Statistical Package for the Social Sciences (SPSS) software (version 20.0) for statistical analysis for quantitative data. 

Strengthening the Reporting of Observational studies in Epidemiology (STROBE) and Consolidated criteria for Reporting Qualitative research (COREQ) guidelines were used to report the study findings (Von et al., 2007; Tong et., 2007).

## Results

A total of 245 participants were included in the study, majority of them 122 (49.7%) were SLT users followed by dual users 71 (28.9%) who used both smoking and SLT. The mean age of the total participants was 38.7±12.7 years, the mean age among smokers, SLT users and dual users were 40.1±12.3, 39.6±13.3 and 35.5±11.9 years respectively. The females (4.9%) reported to use only SLT compared to males (95.1%) who used smoking, SLT and dual use. Majority of the participants belonged to upper lower socioeconomic status among all the three groups (Table 1). 

The evaluation and comparison of level of nicotine dependence among the exclusive smoking and smokeless tobacco users revealed significant difference (p value .01) between the two groups with majority proportion representing low dependency in both the groups. Very high dependence was only reported in participants consuming Smokeless Tobacco (Table 2). Evaluation and comparison of nicotine dependence among the dual tobacco users also revealed significant difference with majority of smoking dependence dual users reporting low dependence whereas majority of smokeless tobacco dual users showing high dependence (Table 3). 

The participants were in the pre-contemplation phase (haven’t thought of quitting) “No Plans Yes, will think of quitting”, contemplation phase (thinking of quitting) “Will quit from today, after cleaning teeth” and action phase (currently quit) “Reduced from New Year, will quit this month”. About 52% of participants had made atleast one quit attempt in the past. 55.8% smokers, 50% SLT users, and 52.1% dual users had made atleast one attempt to quit tobacco in the past. Majority of the participants reported to be in the contemplation phase with 82.7% among smokers, 71.3% SLT users, and 83.1% of dual users respectively. SLT users (12.3%) were reported to have taken action in the past to quit tobacco compared to 9.6% smokers, and 1.4% of dual users. Proportion of past quitting attempts were reported to be higher in participants who were smoking only (55.8%) (Table 4). A comparative evaluation of willingness to quit among different users revealed no significant difference among smokers and SLT users; however, a significant difference (p value 0.038) was reported among the dual users (Table 5). 

Twenty-two sub-themes and five themes were generated from the qualitative data. Plans about quitting tobacco, reasons for planning to quit tobacco, foreseeing the process of quitting tobacco, perceived advantages of quitting tobacco, and tobacco use risk perception were the themes generated.

*Plans about quitting tobacco*

The participants reported that they had never thought about quitting, thinking about quitting, and have taken steps to quit tobacco. This data was categorized in to sub-themes as pre-contemplation phase, contemplation phase, and action phase. 

Pre-contemplation phase: “No Plans yet. Will think about quitting”

Contemplation phase: “Will quit from today, after getting my teeth cleaned”

Action phase: “I have reduced from new year, will quit this month” (Table 6).

*Reasons for planning to quit tobacco*

The various reasons for planning to give up tobacco use were sub-themed as macro environment, micro environment and no reason to quit. The macroenvironment constituted the community, doctor, health care provider (HCP), friends, and family as the influencers to quit tobacco. The microenvironment consisted of concerns over self, factors such as one’s own general health, dental health, self-motivation, esthetics, and spiritual reasons. Some of the participants also reported that there was no reason to quit tobacco (Table 3).

“Cancer can occur, teeth are getting spoiled. Multiple teeth are missing due to tobacco use.”

“I have ulcer in my mouth. Doctor told it is pre-stage of cancer, so I want to quit”

“I have small kids, need to quit for the sake of my family”

“It is harmful for the body and my family wants me to quit”

*Foreseeing the process of giving up tobacco product use*

**Figure 1:**
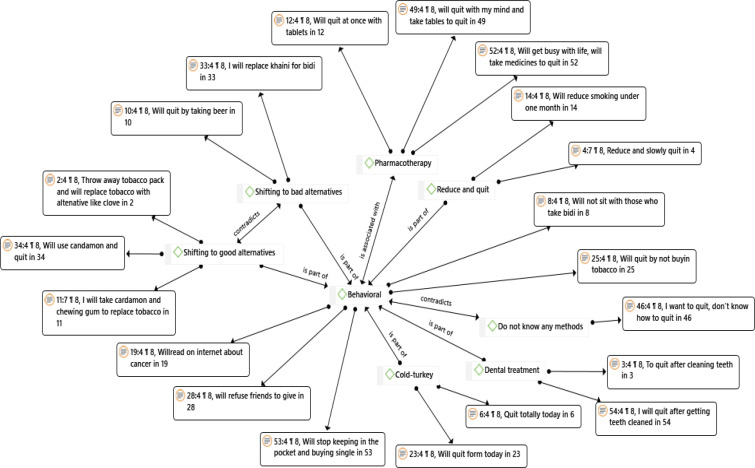
Perception on Different ways to Quit Tobacco

Network analysis (Figure 1) was done to map the participants perception of quitting tobacco. The process of tobacco quitting was coded as behavioral methods, pharmacotherapy, and don’t know how to quit.

Behavioral methods: Behavioral methods reported by the participants were cold turkey i.e., deciding to quit immediately, reducing or tapering tobacco use gradually, not buying tobacco products, stop keeping tobacco in the pocket, reading about the ill effects of tobacco on internet, saying “no” when offered tobacco, and shifting to alternate products. Few participants perceived shifting to alternative like cardamom, clove, fennel seeds will help in quitting and contrastingly some participants perceived shifting to other forms of tobacco and alcohol will help in quitting the current habit; these two sub-themes contradict each other with the type of products used to quit tobacco. 

Pharmacotherapy: Participants were aware of nicotine replacement gums and medicines as a way to quit tobacco. 

However, few participants were not aware of any methods that aided to quit tobacco (Table 6) (Figure 1).

“I will replace khaini with chocolate and cardamom”

“Will stop keeping cigarette in pocket and will buy single cigarette”

“I will try to stop use in one week by slowly reducing the amount of khaini”

*Perceived advantages of quitting tobacco*

Participants believed quitting tobacco can help improving the esthetics, have a better economy, self-esteem, and a better physical and mental health. The esthetic concerns of the participants included better teeth, lips, and color of the skin. Participants felt that quitting tobacco can help them save the money spent on tobacco. The perceived physiological advantages of quitting reported were healthier lifestyle, better oral and general health, and improved stamina. Psychological aspect of having a relaxed mind after quitting was also considered an advantage. One participant had a misconception that only smoking forms of tobacco are harmful, and perceived chewing tobacco to be a safe product (Table 6).

“I can save money, and there will be no bad smell from my mouth”

“I will have better self-esteem in the society. I can save money, have a better health”

“I will get good life. My mouth opening will increase and my life will be back”

“Khaini is less harmful than bidi, only smoke is harmful”

*Tobacco use risk perception*

The tobacco users risk perception reported were coded as death, cancer, dental diseases, systemic diseases, and generalized weakness. Oral and lung cancer were mentioned by few participants. Mouth ulcer, reduced mouth opening, oral malodor, gum diseases, and discolored teeth were the dental complaints by the participants. The participants were also aware of the systemic diseases due to tobacco use; heart and lungs were perceived to be affected by tobacco. Specific lung conditions like chest congestion, cough, tuberculosis, and lung cancer were perceived as risk for tobacco use. Participants also reported that generalized body weakness reduced the overall productivity of the work (Table 6).

“I might develop teeth problems or mouth ulcer as I sleep with gutka in mouth”

“If this habit will be there always, and any disease can occur. Cough, TB, and cancer might develop if I continue”

“My heart problem might worsen; cancer and other diseases can occur”

**Table 1:** Demographic Status of Study Participants (N=245)

		Tobacco users (N=245)		
Variables		Smoking only (N=52) N (%)	SLT use only (N=122) N (%)	Dual users (N=71) N (%)
Mean age		40.1±12.3	39.6±13.3	35.5±11.9
Gender	Male	52 (100)	110 (90.1)	71 (100)
	Female	0	12 (9.8)	0
Socio-economic status	Upper	3 (6.1)	5 (4.1)	2 (2.9)
	Upper Middle	9 (18.4)	19 (15.6)	18 (26.1)
	Lower Middle	14 (28.6)	37 (30.3)	22 (31.9)
	Upper lower	21 (42.9)	46 (37.7)	25 (36.2)
	Lower	2 (4.1)	15 (12.3)	2 (2.9)

**Table 2:** Evaluation and Comparison of Level of Nicotine Dependence among the Exclusive Smoking and Smokeless Tobacco Users

Level of nicotine dependence	Smoking dependence	Smokeless Tobacco Dependence	p value
	(FTND)* (N=52)	(FTND-ST)** (N=122)	
Very Low Dependence	16 (30.7)	18 (14.7)	
Low Dependence	24 (46.1)	46 (37.7)	
Medium Dependence	9 (17.3)	27 (22.1)	.01*
High Dependence	3 (5.7)	26 (21.3)	(s)
Very High Dependence	0	5 (4.0)	

* FTND - Fagerstrom test for nicotine dependence; ** FTND-ST - Fagerstrom test for nicotine dependence – Smokeless Tobacco; (s), Statistically significant

**Table 3:** Evaluation and Comparison of Nicotine Dependence among the Dual Tobacco Users

Level of nicotine dependence	Smoking dependence	Smokeless Tobacco Dependence	p value
	(FTND)* (N=71)	(FTND-ST)**(N=71)	
Very Low Dependence	30 (42.2)	18 (25.3)	
Low Dependence	33 (46.4)	14 (19.7)	
Medium Dependence	4 (5.6)	17 (23.9)	.001*
High Dependence	4 (5.6)	19 (26.7)	(s)
Very High Dependence	0	3 (4.2)	

Dual users- using both smoking and smokeless forms of tobacco; * FTND - Fagerstrom test for nicotine dependence; ** FTND-ST - Fagerstrom test for nicotine dependence – Smokeless Tobacco; (s), Statistically significant

**Table 4:** Evaluation of Willingness to Quit among Study Participants

	Phase	Smoking only (N=52) N (%)	SLT use only (N=122) N (%)	Dual users (N=71) N (%)
Willingness to quit	Pre-contemplation phase	4 (7.7)	20 (16.4)	11 (15.5)
	Contemplation phase	43 (82.7)	87 (71.3)	59 (83.1)
	Action	5 (9.6)	15 (12.3)	1 (1.4)
Past Quit Attempt		29 (55.8)	61 (50.0)	37 (52.1)

**Table 5:** Comparison of Willingness to Quit among Different Users

Willingness to quit	Smoking		p value	SLT Use		p value	Dual User		p value
	Present	Absent		Present	Absent		Present	Absent	
Pre-contemplation phase	4	31	0.308	20	15	0.06	11	24	.038* (s)
Contemplation phase	43	146		87	102		59	130	
Action	5	16		15	6		1	20	

**Table 6:** Themes and Sub-themes on Tobacco Quitting Perception

Theme	Sub-theme
Plans about quitting tobacco	Pre-contemplation phase
	Contemplation phase
	Action
Reasons for planning to give up use of tobacco product	Macroenvironment
	Microenvironment
	No reason
Foresee the process of quitting tobacco	Behavioral
	Cold-turkey
	Tapering tobacco use
	Shift to good alternatives
	Shift to bad alternatives
	Pharmacotherapy
	Don’t know
Perceived advantages of quitting tobacco	Esthetic
	Economic
	Physiological
	Psychological
Risk perception of tobacco use	Death
	Cancer
	Systemic health
	General weakness
	No problem

## Discussion

This study used a mixed-method model to determine the tobacco dependence and tobacco quitting perception among the individuals visiting the dental hospital in India. This study provides both qualitative and quantitative data which helps the tobacco researchers to understand the quitting perception among tobacco users. 

In the current study 83% of the participants from the current study showed interest in quitting tobacco. In a study conducted among the Aboriginal and Torres Strait Islander 70% participants were willing to quit (Nicholson et al., 2015). Patil et al., (2015) reported that 88.9% of the tobacco users visiting the dental hospital were planning to quit the use. According to the GATS-2 India survey only 38.5% of smokers and 33.2% SLT users attempted to quit in the past (GATS 2, 2017). However, in the present study only 12.3% SLT users, 9.6% smokers, and 1.4% of dual users reported to have attempted to quit tobacco.

The study participants were in either the contemplation, pre-contemplation and action phases. Behavioral, pharmacotherapy, shift to alternatives were reported by the study participants as methods to quit tobacco. The participants reported reducing the tobacco use gradually, not keeping tobacco in pocket, and buying single tobacco as ways to quit tobacco. Similar findings were reported in a focus group discussion among the college students, not buying cigarettes and reducing cigarette consumption over time, were markers of a quit attempt (Berg et al., 2013). Veeraiah et al., (2020) reported that tobacco users have substituted chocolate, bubble gum and tulsi (basil) leaves to overcome craving, similarly the present study participants have reported Indian spices such as clove, cardamom, and chewing gum as alternatives to quit tobacco. 

In the current study the macro environment consisting of doctor, dentist, family and society influenced the tobacco users to quit, and the participants were concerned about their teeth esthetics due to tobacco use. In contrast, a study conducted among the Indian women, Schensul et al., (2018) reported most participants thought that the best way to quit was on their own and only 12% mentioned taking help from a health care provider. Dhumal et al., (2014) reported that tobacco quit intentions were significantly higher among those who were advised by doctors or health care providers (HCP) compared to non-advised participants, similarly the present study participants also reported dentist advise as a reason to quit tobacco. Evidence supports that individual visiting the dental hospital had a positive perception of the role of dentists in smoking cessation activities (Sood et al., 2014). Dentists can have a positive impact on the tobacco users by providing a brief advice to quit tobacco during the routine dental procedures.

Rana et al., (2021) reported increased perception of quitting smoking with increase in the price of smoked tobacco products, the current study participants also perceived saving money spent on tobacco as an advantage of quitting tobacco. Anti-tobacco messages have been reported to have a positive influence on tobacco user’s intentions to quit (Surani et al., 2012). However, none of the current study participants mentioned anti-tobacco messages as an influence to quit tobacco.

The current study participants were aware of the health risks of tobacco use; death, cancer, dental diseases, systemic diseases, and generalized weakness were the overall tobacco risk perceptions drawn from the qualitative data collected. However, this was in contrast to the findings of Kakodkar and Bansal (2013), where majority participants were unaware of health effects, and only one-fourth believed that hookah smoking is harmful to health.

Limitations to this study are firstly, limited generalizability of the findings due to the recruitment of participants, from the dental outpatient department only. Secondly, the participants reported to tobacco cessation center were those referred by other department dentists, there might be few tobacco users who received brief advice from other dentist and did not report to us at the tobacco cessation center, whose quitting perceptions might be missed in this study. Additionally, bias due to the self-report nature of the assessments, and few participants hurried during the interview as they wanted to go for their respective dental treatments. Finally, it is possible that we failed to tap some dimensions of the tobacco cessation perceptions; further research involving patients at medical or community set-up is required to make certain profound assessments of such dimensions.

The smokers, SLT users and dual users were willing to quit tobacco and they were aware of the disadvantage of using tobacco and advantages of quitting tobacco. The macro environment consisting of doctor, dentist, family, and society influenced the tobacco users to give up tobacco use. Some participants were ignorant about the ill-health effects of tobacco and considered SLT products to be safe. It is important that the dental professionals understand the different risk perceptions of the tobacco users and provide tailored tobacco cessation. There is a need to increase the health awareness campaigns and sensitize the tobacco users about the existing tobacco cessation services.

## Author Contribution Statement

Study conception and design: Priyanka Ravi, Anupama Ivaturi, Harsh Priya; Data collection: Priyanka Ravi, Anupama Ivaturi, Monica Dev; Analysis and interpretation of results: Priyanka Ravi, Anupama Ivaturi, Diptajit Das, Charu Khurana; Draft manuscript preparation: Priyanka Ravi, Upendra Singh Bhadauria, Charu Khurana; Draft revision and final approval: Priyanka Ravi, Anupama Ivaturi, Harsh Priya. All authors reviewed the results and approved the final version of the manuscript.

## Ethical conduct of research

The study was approved by the Institute Ethics Committee, All India Institute of Medical Sciences, New Delhi (Ref. No.: IEC-48/07.02.2020).

## Availability of data

Data can be obtained from the corresponding author, Dr. Harsh Priya, Associate Professor, Department of Public Health Dentistry, Centre for Dental Education and Research, All India Institute of Medical Sciences, New Delhi, India. Pincode-110029. e-mail address: drharshpriya@gmail.com

## Statement conflict of interest

The authors reported no potential conflict of interest.
